# Unveiling the Role of Formulation and Process Variables in Nanoemulsion Preparation: A Data-Driven Approach Using High-Energy Ultrasonication

**DOI:** 10.3390/pharmaceutics18070786

**Published:** 2026-06-26

**Authors:** Diego Romano Perinelli, Ledjan Malaj, Laetitia Novelli, Marco Cespi, Giulia Bonacucina

**Affiliations:** 1 Chemistry Interdisciplinary Project (ChIP) Research Center, School of Pharmacy, University of Camerino, 62032 Camerino, Italy; diego.perinelli@unicam.it (D.R.P.); laetitia.novelli@unicam.it (L.N.); giulia.bonacucina@unicam.it (G.B.); 2 Department of Pharmacy, University of Medicine Tirana, 1005 Tirana, Albania; ledjan.malaj@umed.edu.al

**Keywords:** oil viscosity, ultrasonication, droplets size, surfactants, dynamic light scattering, artificial neural network

## Abstract

**Background:** Oil-in-water nanoemulsions (NEs) represent versatile platforms for the delivery of hydrophobic compounds and find a wide range of applications in different fields such as food, cosmetics, agriculture, pharmaceutics, and oil and gas industries. Various methodologies can be applied for the preparation of NEs as low-energy and high-energy methods. Among them, high-energy ultrasonication (HEU) is a popular technique in research laboratories or small manufacturing facilities. However, a clear gap remains in understanding how, and to what extent, experimental parameters and the chemical and physical characteristics of the components affect the formation and properties of NEs through HEU. **Methods:** In this work, a comprehensive screening of factors (oil viscosity and density, surfactant type, processing parameters, and formulation composition) affecting NEs formation and quality was performed and an artificial neural network (ANN) was applied to determine the relative relevance of each parameter. **Results:** Oil viscosity revealed to be the primary factor affecting droplet size (Z_avg_) and polydispersity index (PDI), with high-viscosity oils leading to poor emulsification into nanosized droplets. Higher processing temperatures improved NE formation by reducing viscosity during sonication. Ultrasound amplitude and pulse mode influenced NE characteristics, particularly under challenging conditions. Surfactant type and oil content had, instead, minor effects on the NEs’ features. ANN modelling accurately predicted NEs’ properties and identified critical viscosity limits for successful nanosized emulsification (Z_avg_ < 300 nm and PDI < 0.4). **Conclusions:** These findings provide a predictive basis for rational NE design under HEU, serving as a guide for researchers working in different fields.

## 1. Introduction

Nanoemulsions (NEs) are kinetically stable nanometric dispersed systems consisting of two immiscible liquid phases, typically referred to as a water phase and an oil phase, stabilized by one or more surface-active compounds. The internal phase, present in smaller quantities, is dispersed as nanometric droplets, while the surfactant content remains lower than that of the internal phase (with a surfactant-to-oil ratio, SOR, of around or less than 1) [1,2].

NEs are widely studied and utilized in various sectors. Among these, the pharmaceutical field is prominent, with O/W NEs commonly employed as carriers for delivering hydrophobic drugs or contrast agents. In recent years, however, these systems have also gained attention as an attractive platform in industries such as food, cosmetics, agriculture, and even oil and gas industries. The broad range of applications for NEs is extensively described in the literature [3,4,5,6,7,8].

NEs can be prepared using various methodologies, which are categorized into two main groups: low-energy and high-energy methods. Low-energy methods involve the initial preparation of an emulsion, which is then converted into an NE by modifying either its composition (emulsion inversion point, EIP) or temperature (phase inversion temperature, PIT) [2,3]. Despite the apparent simplicity and low cost of these methods, they are not commonly used due to the need for strict control over the type and quantity of components, particularly surfactants [9]. High-energy methods are the most commonly employed for NE preparation. These methods involve the initial preparation of an emulsion, which is then processed by forcing the fluid through a narrow gap using high-pressure homogenizers or microfluidizers (high-pressure homogenization, HPH). Alternatively, the droplet size of the emulsion can be reduced using high-energy shock waves that generate turbulence through cavitation, as in high-energy ultrasonication (HEU) [2,3]. Both HPH and HEU are widely used; HPH is more suitable for processing large quantities of materials due to its higher production capacity, despite the higher cost of the equipment. In contrast, HEU is ideal for producing small batches, up to a few milliliters, and is significantly more affordable. These features, in addition to the compact size of ultrasonicators and their versatility (e.g., they can be used to produce other colloidal systems, as cell disruptors, or as processing aids in extraction processes), make HEU a popular technique for NE preparation in research laboratories and small manufacturing facilities. Despite its popularity and widespread use, there is an evident lack of knowledge regarding how, and to what extent, experimental conditions as well as the chemical and physical properties of the components influence the preparation of NEs. Theoretical descriptions of NE formation were first proposed by Hinze [10] and later modified by Gupta et al. [11]. While these models are valuable for understanding how to control the final droplet size, they remain theoretical frameworks that are challenging to apply in everyday practice due to the need to determine dimensionless parameters, such as the Weber number and the Ohnesorge number. These parameters depend on several physical variables, including localized inertial forces, energy dissipation rates, and surface tension gradients, which are often difficult to obtain or measure in a standard laboratory setting. In contrast, the number of scientific publications focused on experimentally quantifying the relevance of both processing parameters and formulation components is limited, and, in some cases, lacks general applicability. Kentish et al. [12] evaluated the effect of power input and ultrasonication time on the droplet size of NEs prepared with flaxseed oil and polysorbate 40, while Alzorqi et al. studied the effect of water content, oil-to-surfactant ratio, ultrasonic power, and processing time on the preparation of NEs containing palm olein as the oil phase and a mixture of polyoxyl 40 hydrogenated castor oil and oleoyl macrogol-6 glycerides as the surfactant [13]. The same parameters were also assessed by Guzmán in Passiflora edulis seed oil NEs stabilized with a blend of Tween 80/Span 85 [14]. Miastkowska et al. focused their attention on the effect of surfactant type and amount in preparing NEs with thistle oil [15]. The main limitation of these studies is that they evaluate a limited number of parameters and do not consider the characteristics of the oil phase, thus lacking general applicability.

Our study aims to address these limitations and bridge the gap by providing a comprehensive understanding that is currently lacking. It is crucial to conduct a study that considers a broader range of parameters, ensuring greater general applicability. Specifically, our work will consider a multitude of parameters such as the initial emulsion droplet size (emulsification conditions), ultrasonication parameters (processing time, processing temperature, ultrasound amplitude, and on/off pulse ratio), as well as the effects of density and viscosity of the oil phase (with six different oils selected), different surfactants (three different amphiphiles tested), and the oil–surfactant ratio. All the parameters were evaluated in tailored experiments to identify their specific relevance. Subsequently, all parameters were analyzed collectively using an artificial neural network (ANN) to determine their relative importance and identify the critical factors. Leveraging the ANN’s predictive capability, it was possible to calculate the limit values of the critical variables necessary for optimal NE preparation.

## 2. Materials and Methods

### 2.1. Materials

Ethyl oleate (Crodamol EO, Croda, Cowick Hall, UK), isopropyl myristate (ACEF, Fiorenzuola D’Arda, Italy), medium chain triglycerides (Myritol 318, BASF, Ludwigshafen, Germany), refined sesame seed oil (ACEF, Fiorenzuola D’Arda, Italy), extra virgin olive oil (Oleificio RM spa, S. Alessio, Italy), paraffin oil (mineral oil FU, ACEF, Fiorenzuola D’Arda, Italy), and silicone oil (Abil 100 o Acesil 350, ACEF, Fiorenzuola D’Arda, Italy) were the oil phase components. The surfactants used were the polysorbate 80 (ACEF, Fiorenzuola D’Arda, Italy), ceteareth 20 (Brij CS20), and caprylyl/capryl glucoside (Plantacare 810 UP, BASF, Ludwigshafen, Germany). Demineralized water was prepared with a reverse osmosis system (RO 60 TS-demi2, GAMMA 3 s.n.c., Castelverde, Italy). All the materials used throughout this study were at least of cosmetic grade.

In the manuscript, the oils and surfactants are abbreviated as follows: Et for ethyl oleate; IsoM for isopropyl myristate; MCT for medium chain triglycerides; EvO for extra virgin olive oil; SeO for refined sesame seed oil, PaO for paraffin oil; SiO for silicone oil; P80 for polysorbate 80; CS20 for ceteareth 20; and CCG for caprylyl/capryl glucoside.

### 2.2. Methods

#### 2.2.1. Characterization of Oils

The density of the oils (Et, IsoM, MCT, EvO, SeO, PaO, and SiO) was measured using a digital density meter with an oscillating U-tube (DA-100M, Mettler Toledo, Schwerzenbach, Switzerland) at 25 °C. Flow curves of all oils were determined at 25 °C in the shear rate range of 0.1 s^−1^ to 100 s^−1^ using a rotational rheometer (Kinexus Lab+, Malvern, UK) fitted with a CP4/40 cone-plate geometry. The resulting shear stress vs. shear rate data were evaluated using the power law model:

σ = PLV∙D^PLI^

where σ is the shear stress, D the shear rate, PLV the power law viscosity (or consistency index), and PLI the power law index (or flow index). Each sample was analyzed in triplicate.

#### 2.2.2. Nanoemulsions Composition

In most experiments, unless otherwise specified, the nanoemulsions were formulated with an oil phase at 6% *w*/*w* and 2% *w*/*w* P80 as the surfactant (resulting in a surfactant-to-oil ratio, SOR, of 0.33) following previous work on NEs prepared with HPH [16,17]. The oil phase consisted of all seven types of oil previously characterised (EtO, IsoM, MCT, EvO, SeO, PaO, and SiO).

Additional experiments were conducted by modifying the oil content (6–10% *w*/*w*) and/or the surfactant type (P80, CS20, and CCG), while either maintaining a constant surfactant concentration (2% *w*/*w*) or keeping the SOR constant at 0.33.

Unless otherwise specified, all formulation composition percentages are expressed as *w*/*w*.

In this study, the different oil phases are also referred to simply as oil for the sake of brevity and readability.

#### 2.2.3. Nanoemulsions Preparation

All the nanoemulsions (NEs) were prepared using a two-step procedure. First, the oil was added to the aqueous phase (containing water and surfactant) to form an emulsion. Subsequently, this emulsion was subjected to ultrasonic processing to obtain a NE.

All preliminary emulsions were prepared by dissolving the surfactant in an appropriate amount of water (determined by the oil and surfactant concentrations) in a 50 or 100 mL beaker under magnetic stirring. Subsequently, the oil was added to the beaker and emulsified using a high-energy rotor–stator disperser (Ultra-Turrax T25 basic, IKA^®^ Werke GmbH & Co. KG, Staufen, Germany). The emulsification process was conducted using an 8 mm-diameter rotor operating at 9500 rpm (corresponding to a linear velocity of approximately 8 m/s) for 5 min. Additional tests were performed by varying the operating time (5–15 min) and rotor speed. In the latter case, linear velocities ranging from 8 to 27 m/s were achieved by adjusting the rotor angular velocity (6500–21,500 rpm) and/or the rotor size (8 and 12 mm).

NEs were then prepared by processing 40 g of the preliminary emulsions using a 500 W ultrasound sonicator (Fisherbrand Model 505, Fisher Scientific Italia, Segrate, Italy) operating at a frequency of 20 kHz. The sonicator featured a microtip probe with a diameter of 1/4″ (6.3 mm) screwed into the threaded end of the standard 1/2″ probe. This configuration is suitable for processing volumes of 5 to 50 mL and capable of applying a maximum amplitude of 170 µm. According to the manufacturer, the 1/4″ microtip is recommended to operate at a maximum amplitude level of 50%. However, preliminary trials with 40 g of water in a 50 mL beaker showed that an amplitude of 50% and 40% caused splashing, with 30% being the highest usable amplitude.

The initial ultrasonication conditions applied included an amplitude of 30%, a pulse mode of 10/10 (10 s of ultrasound operation alternated with 10 s off), ambient temperature, and processing times of 1, 3, 6, and 9 min. Additional tests were conducted by reducing the amplitude to 20% (the lowest applicable level), modifying the pulse mode (10/20, 10/30, and 15/10), or operating in an ice bath (initial temperature approximately 5 °C). The temperature was monitored at the beginning, during, and at the end of each NE processing run using a non-contact temperature sensor (EL301HT-X, Calex Electronics Limited, Leighton Buzzard, UK) connected to a Multiset point Controller (DRR245, Pixsys Electronics, Mellaredo di Pianiga, Italy).

#### 2.2.4. Preliminary Emulsions Characterization

The preliminary emulsions were characterized based on their droplet size distribution. Images of the samples were acquired using an optical microscope (MT9000, Meiji Techno Co., Ltd., Saitama, Japan) equipped with a 3-megapixel CMOS camera (Invenio 3S, DeltaPix, Smoerum, Denmark). The camera acquisition software (DeltaPix Viewer v1.14.11, DeltaPix, Denmark) was calibrated using a graticule optics slide (S22 StageMic 2 mm/0.01 mm, Graticules Optics Ltd., Tonbridge, UK) prior to use. Image analysis was performed using the software ImageJ (v. 1.54J; U.S. National Institutes of Health, Bethesda, MD, USA) following this procedure: the images were calibrated, converted to an 8-bit black-and-white format, and pre-processed by adjusting brightness and contrast levels. The threshold was then set using the Default algorithm and droplets were analyzed with the “Analyze Particles” module, setting a lower size limit of 3 µm to exclude undefined droplets or dust particles and applying a shape filter (circularity higher than 0.7) to remove unseparated droplets, droplets at the image border, or other non-droplet objects (e.g., the calibration bar).

For each sample, at least 1000 droplets were analyzed and the volume droplet size distribution histogram was built using the measured Feret diameters. The median value (D50) and interquartile range (IQR) were calculated to provide a synthetic description of the size distributions.

#### 2.2.5. Nanoemulsions Characterization

All the prepared NEs were initially evaluated through visual inspection and observed using an optical microscope (MT9000, Meiji Techno Co., Ltd., Saitama, Japan). Microscopy evaluation was not intended to measure nanoscale droplets or even visualize them. Instead, it was deployed exclusively as a rapid, direct screening tool to check for the presence of large, microscale oil droplets, macro-aggregation, or phase separation.

Droplet size measurements were performed using dynamic light scattering (DLS) with a Zetasizer NanoS (Malvern Instruments, Malvern, UK), equipped with a backscattered light detector operating at an angle of 173°. For each measurement, 1 mL of the sample was placed in disposable cuvettes and analyzed at a controlled temperature of 25 °C. To ensure accurate and consistent results, the samples were equilibrated at the set temperature for 180 s prior to measurement. The harmonic intensity averaged particle hydrodynamic diameter (Z-average, Z_avg_) and the polydispersity index (PDI), which represent the central tendency of particle size and the breadth or uniformity of the distribution respectively, were used to summarize the size distributions.

The PDI was interpreted as follows: values lower than 0.4 indicate samples with low to moderate polydispersity, while values between 0.4 and 0.7 suggest high polydispersity [18]. Values greater than 0.7 indicate a very broad size distribution, which makes the sample unsuitable for DLS analysis [19]. Consequently, Z_avg_ values for samples with a PDI higher than 0.7 are considered unreliable and were excluded from the analysis.

Each sample was analyzed in triplicate (three independently prepared batches).

#### 2.2.6. Statistics

All data are reported in terms of average and standard deviation unless otherwise specified.

Correlation analyses (Pearson and/or Spearman) were performed using GraphPad Prism V6.01 (GraphPad Software Inc., Boston, MA, USA), with a significance level set at 5%. When the correlation coefficients were found to be statistically significant, their absolute magnitudes were interpreted based on the following criteria: 0.9 < r < 1 indicating a very strong correlation; 0.7 < r < 0.89 for a strong correlation; 0.4 < r < 0.69 representing a moderate correlation; 0.1 < r < 0.39 for a weak correlation; and r < 0.09 indicating a negligible correlation [20,21].

#### 2.2.7. Modelling of Nanoemulsions Preparation

A preliminary analysis was conducted on the PDI data to identify the most suitable modelling procedure. Specifically, the multilinear regression (MLR) approach, starting with a full quadratic model, was compared with an artificial neural network (ANN) multilayer perceptron (MLP). The best MLR model was derived using a stepwise regression procedure, which added or removed terms from the model until all variables not included had *p*-values greater than 0.15. The optimal MLP model was instead selected by performing online training, with variations in the partition ratio between randomly assigned training and testing samples (70/30, 80/20, and 90/10) and testing different network architectures.

After the preliminary analysis, the MLP model was selected and applied to the Z_avg_ outcome as well. All experiments where the HEU process produced samples with a PDI larger than 0.7 were excluded from the Z_avg_ modelling.

MLR was carried out using Minitab^®^ V 18.1 (Minitab LLC, State College, PA, USA), while the MLP was implemented using SPSS^®^ V 28.0 (IBM Corp., Armonk, NY, USA).

## 3. Results and Discussion

### 3.1. Experimental Evaluation of Parameter Relevance

#### 3.1.1. Oil Phase Properties and Emulsification Conditions

The starting-point composition for NEs consisted of an oil phase at 6% and 2% P80 as the surfactant, chosen for its effectiveness when processed using HPH [17]. These formulations were prepared following the “initial” HEU conditions detailed in Section 2.2.3. During the first round of experiments, these conditions were held constant, except for the oil phase, where all seven selected oils were tested. As a preliminary characterization, all oils were analyzed in terms of rheological behavior and density. While all systems exhibited nearly Newtonian behavior (PLI around 1), they differed in viscosity (SiO > PaO > EVO ≈ SeO > MCT > IsoM > EtO) and density (SiO > MCT > EVO ≈ SeO > PaO ≈ EtO > IsoM), as illustrated in Supplementary Figure S1.

**Figure 1:**
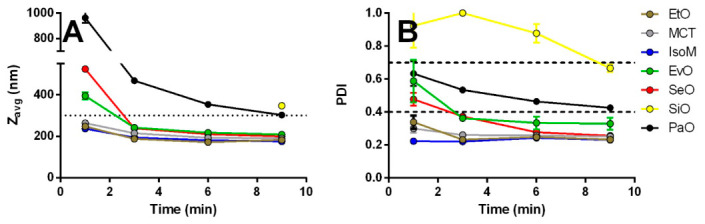
Effect of oil type on Z_avg_ (**A**) and PDI (**B**) of NEs prepared following the “initial” HEU conditions (composition oil phase at 6% *w*/*w* and 2% *w*/*w* P80 as the surfactant, preliminary emulsion prepared operating at a linear velocity of approximately 8 m/s for 5 min, NEs prepared with ultrasonication with an amplitude of 30%, a pulse mode of 10/10 and at ambient temperature). Each value on the plot represents the average and standard deviation of 3 replicates.

All the prepared NEs were analyzed in terms of size (Z_avg_) and droplet size variability (PDI). As shown in Figure 1, the choice of the oil phase had a significant impact on the NEs features. Specifically, highly polydisperse NEs were obtained using SiO, but only after prolonged processing times (9 min). At shorter processing time, the polydispersity was so high (PDI > 0.7) that the measured Z_avg_ was unreliable. In contrast, NEs prepared with other oil phases were always successfully obtained, displaying notable differences in Z_avg_ and PDI, particularly at shorter processing times. With oil phases such as EtO, IsoM, and MCT, the NEs exhibited moderately low polydispersity and small Z_avg_ (<300 nm). Conversely, NEs prepared with PaO were characterized by high polydispersity and larger Z_avg_ (>300 nm at all processing times). For SeO and EVO, the observed characteristics varied between these two extremes, depending on the processing time. Based on these findings, the oil phases can be classified according to their suitability for NE preparation: EtO, IsoM, and MCT were the most favorable, yielding NEs with a relatively small Z_avg_ and moderately polydisperse droplet size; PaO showed limited suitability, producing large and highly polydisperse NEs; SeO and EVO exhibited intermediate behavior, with performance depending on processing time; SiO was the least suitable, as NEs only formed after prolonged processing times.

**Figure 2:**
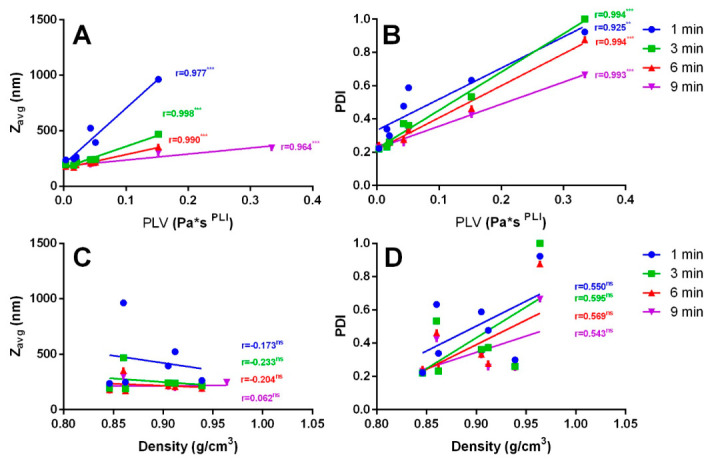
Pearson correlation analysis between the oil viscosity and Z_avg_ (**A**) and PDI (**B**), and between the oil density and Z_avg_ (**C**) and PDI (**D**) of NEs prepared following the “initial” HEU conditions (composition oil phase at 6% *w*/*w* and 2% *w*/*w* P80 as the surfactant, preliminary emulsion prepared operating at a linear velocity of approximately 8 m/s for 5 min, NEs prepared with ultrasonication with an amplitude of 30%, a pulse mode of 10/10 and at ambient temperature). The density and PLV values for all the oils are reported in Figure S1 of the Supplementary Materials. Asterisks indicate the statistical significance of the Pearson correlation coefficient (** *p* < 0.01, *** *p* < 0.001; ns: not significant).

To assess whether oil density or viscosity were relevant factors in NE preparation, a correlation analysis was conducted between these properties (density and viscosity) and the resulting NE features Z_avg_ and PDI. The results, presented in Figure 2, clearly demonstrated that both Z_avg_ and PDI strongly depend on oil viscosity (Pearson r consistently above 0.9 and statistically significant, indicating a very strong correlation, Figure 2A,B). In contrast, no statistically significant correlation was observed with oil density (Figure 2C,D).

Interestingly, the oil type affected not only the NEs but also the preliminary emulsions. Indeed, the emulsion droplet size (median Feret diameter D50) varied from approximately 30 to 150 µm as a function of the oil phase used (Figure S2). Correlation analysis confirmed a strong, statistically significant relationship between D50 and oil viscosity (Pearson r = 0.74), while no correlation was found with density (Figure S3). Additionally, a statistically significant correlation was identified between the D50 of preliminary emulsions and the resulting Z_avg_ and PDI of the NEs (Figure S4). It should be noted that these correlations (involving D50) were weaker than those reported in Figure 2.

**Figure 3:**
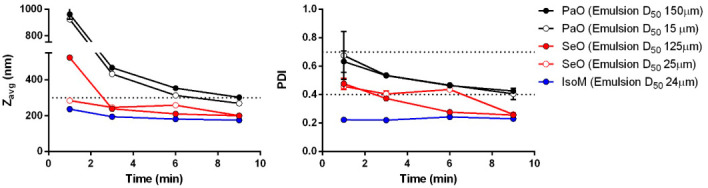
Effect of the droplet size of preliminary emulsions on Z_avg_ (on the **left**) and PDI (on the **right**) of NEs prepared with IsoM, SeO, and PaO following the “initial” HEU conditions. Each value on the plot represents the average and standard deviation of 3 replicates.

These results raised the question of whether the influence of oil viscosity on the NEs was a direct result of its effect on the ultrasonication process or an indirect consequence of the varying droplet sizes in the emulsions prior to ultrasonication. The latter scenario implies that smaller droplet sizes in the preliminary emulsion led to smaller Z_avg_ in the NEs. To clarify this point, three oil phases with differing abilities to form NEs (IsoM, SeO, and PaO) were selected. The preliminary emulsification conditions (time, speed and/or rotor size) were adjusted to achieve nearly comparable emulsion droplet size distributions, with a D50 of approximately 20 μm and a D75 below 30 μm (Figure S5). The NEs were then prepared using the same parameters as those applied for NEs in Figure 1, evaluating 1, 3, 6, and 9 min of processing time. The Z_avg_ and PDI results of the NEs prepared from emulsions with comparable droplet size are reported in Figure 3 along with those previously obtained under standard emulsification conditions. These data clearly indicate that the emulsification process (time, speed, and/or rotor size) does not influence the subsequent NE preparation. Thus, it can be stated that oil viscosity directly affects the features of NEs during ultrasonication, regardless of the droplet size of the preliminary emulsion, and, more generally, that viscosity appears to be a critical parameter for obtaining NEs.

#### 3.1.2. Ultrasonication Parameters

The second round of experiments focused on evaluating HEU processing parameters, including the presence of ice, the amplitude level, and the pulse ratio, and their effects on the characteristics of the NEs.

**Figure 4:**
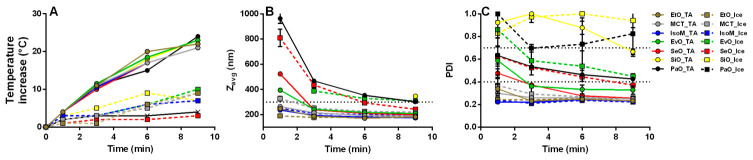
Effect of temperature control (ambient temperature or ice bath) on temperature increases (**A**), Z_avg_ (**B**), and PDI (**C**) of NEs prepared following the “initial” HEU conditions. Each value on the plot represents the average and standard deviation of 3 replicates.

The use of an ice bath (or alternative cooling methods) during NE preparation is a common practice to mitigate heat generation during processing [12,22,23]. NEs were prepared with all oils following the “initial” HEU conditions, operating under both ambient and ice bath conditions. First, the effect of the ice bath was evaluated in terms of temperature increases during NE preparation, as reported in Figure 4A. The presence of ice had a marked effect, limiting temperature increases to a maximum of approximately 10 °C for the longest processing time (9 min). Conversely, without the use of an ice bath, the temperature rose to over 20 °C at 9 min. Comparable temperature increases with or without ice were observed exclusively for 1 min of processing time. No evident differences in terms of temperature increases related to the type of oil phase were observed. Additionally, the effect of the ice bath was evaluated in terms of the resulting NEs’ features. The use of an ice bath had a noticeable effect on the dispersed phase of NEs prepared. Specifically, it led to an increase in both droplet Z_avg_ and PDI. This effect was particularly evident in NEs prepared with higher viscosity oils such as SeO, EVO, PaO, and SiO (Figure 4B,C), making such conditions unsuitable for NE preparation in some cases (e.g., SiO and PaO at all processing times, and EVO at shorter processing times). These results can be explained by considering the findings from the previous section on oil features (Section 3.1.2). If oil viscosity is a highly relevant parameter for NE preparation, it follows logically that temperature, which affects viscosity, also plays a significant role. The positive effect of higher temperatures on NE preparation has been described in previous studies [24,25], although the results of the present study provide further clarification and explanation.

To evaluate an alternative procedure for temperature control, different on/off ratios in pulse mode were tested. As the previous results indicated no effect of the oil type on temperature increases, this set of experiments was performed under “initial” HEU conditions using EtO as the oil phase. Increasing the off phase to twice the duration of the on phase (pulse 10:20) resulted in a temperature increase of approximately 17–18 °C, while extending it up to three times (pulse 10:30), limited the heating to 12–13 °C. This latter thermal rise is nearly comparable to the results obtained with the ice bath procedure. Adjusting the pulse ratio has the advantage, contrary to the use of an ice bath, of starting the ultrasound processing at ambient temperature and avoiding the negative effects of low temperatures on viscosity. However, using a 10:30 ratio doubles the total processing duration (from 18 min with a 10:10 ratio to 36 min with a 10:30 ratio). Another interesting result was observed when setting the pulse ratio to 20:10 while operating under ice bath conditions. In this case, the temperature increase was nearly comparable to that observed with the standard 10:10 ratio, but the processing time was reduced by approximately 25% (from 18 min to 13.5 min). The effect of the pulse ratio on the temperature increases is reported for an ethyl oleate NEs in Supplementary Materials (Figure S6).

**Figure 5:**
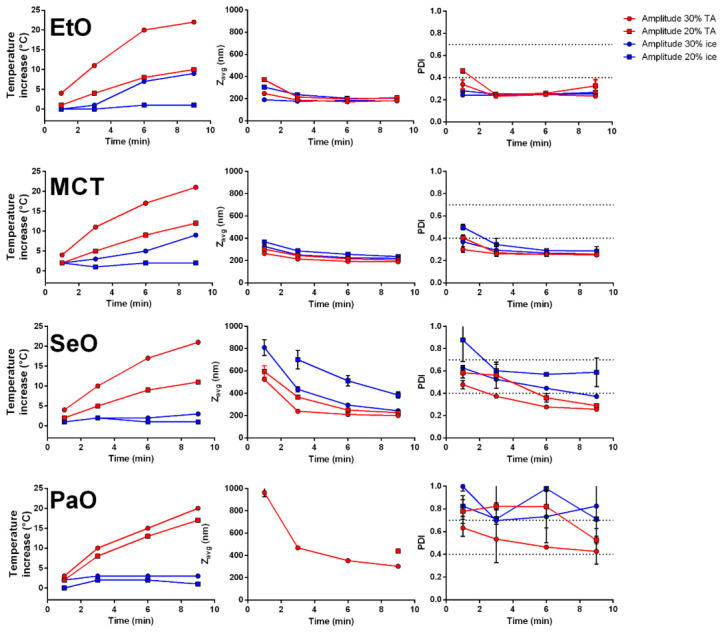
Effect of temperature control (ambient temperature or ice bath) and ultrasound amplitude on temperature increases (**left panel**), Z_avg_ (**middle panel**), and PDI (**right panel**) of NEs prepared following the “initial” HEU conditions using EtO, MCT, SeO, and PaO as oil phase. Each value on the plot represents the average and standard deviation of 3 replicates.

Finally, the effect of the ultrasonicator amplitude was assessed. In this set of experiments, only two amplitude levels were tested, 30% and 20%, as higher amplitudes with the 1/4” microtip probe caused splashing in the samples, while 20% represented the lowest operational limit. Four oil phases were selected based on their viscosity/NEs formation ability: EtO, MCT, SeO, and PaO. Preparations were carried out at both ambient temperature and under ice bath conditions, with the results reported in Figure 5. Reducing the amplitude from 30% to 20% markedly affected the increase in processing temperature. Under ambient conditions, operating at 20% amplitude with a 10:10 pulse ratio, led to a temperature increase almost comparable to those obtained operating at 30% amplitude with a pulse ratio of 10:20 or 10:30. In contrast, when using an ice bath, the lowest amplitude almost completely avoided any increment of temperature. From a general point of view, the reduction in amplitude affected the NEs features but in a manner dependent on the oil viscosity. For low-viscosity oils (EtO and MCT), a lower amplitude did not result in a marked change in Z_avg_ and PDI. In contrast, for the high-viscosity PaO, NEs could not be prepared at 20% amplitude, except for the sample processed for 9 min at ambient temperature, which exhibited broad polydispersity. In the case of SeO, NE features were markedly affected by the amplitude level, with the effect on the Zavg and PDI depending on processing time and the presence or absence of the ice bath.

The effect of ultrasound power, expressed here as amplitude (%), is often reported in the literature as acoustic power (W). It is important to emphasize that amplitude and acoustic power are not the same. Specifically, amplitude refers to the oscillation range of the probe tip that generates the ultrasonic wave, whereas power indicates the energy required (electric current consumption) to achieve a specific amplitude. A constant ultrasonic wave amplitude can be obtained only by directly setting the amplitude, rather than the power. In fact, at constant power, the amplitude of the ultrasonic wave depends on the properties of the processed material (since different materials require different power levels to reach the same amplitude). Despite the differences in their definitions and meanings, both terms relate to the energy transmitted by ultrasound and can be used for general comparison of results. In this context, the literature consistently shows that increasing the energy input produces NEs with smaller droplet sizes [26,27,28], as confirmed by the present work. The novelty of the results reported here lies in the emergence of parameter interactions, specifically the variation in ultrasound energy leading to different effects on NEs as a function of processing temperature, time, and oil viscosity.

#### 3.1.3. Surfactant Type

Surfactants are crucial components in NE preparation, as they promote the dispersion of the two immiscible phases and ensure the stability of the preparation over time. In all previous experiments, polysorbate 80 (P80) was used because it is one of the most commonly employed amphiphiles for O/W dispersion systems [2,6,26] and is also known for its efficacy [17]. To assess the effect of different surfactants, further experiments were carried out to compare P80 with ceteareth-20 (CS20) and caprylyl/capryl glucoside (CCG), all at the concentration of 2%. The selection of these three chemically diverse surfactants was based on their identical HLB value (≈15), which was chosen because it represents the HLB of polysorbate 80, identified by literature analysis (2009–2019) as the most widely used surfactant of nanoemulsion formulations [2]. Following our previous work, while surfactants with HLB ≈ 15 show similar efficiency in the initial droplet size reduction during high-energy preparation, they differ significantly in long-term stability [17].

**Figure 6:**
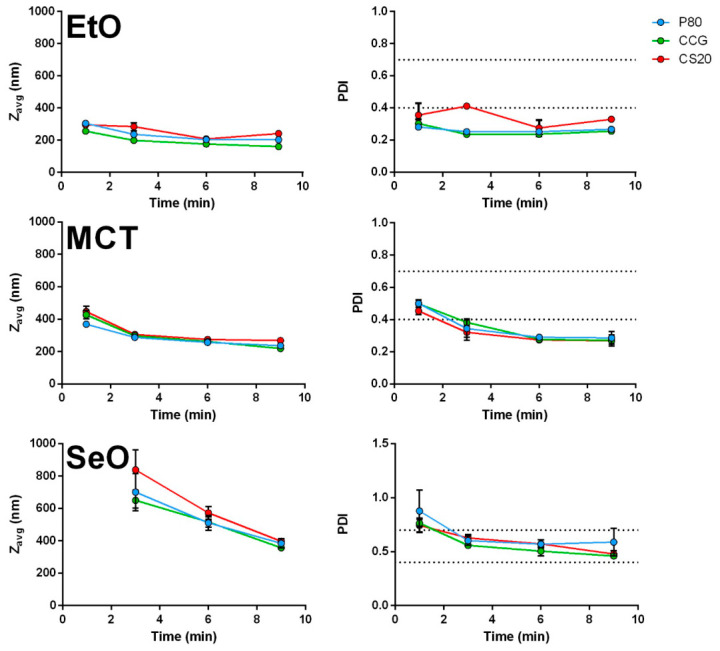
Effect of surfactant type at a constant concentration of 2% on droplet Z_avg_ and PDI of NEs prepared with three different oils, EtO (**upper panels**), MCT (**middle panels**), and SeO (**lower panels**), according to the following conditions: oil phase at 6% *w*/*w*, preliminary emulsion prepared operating at a linear velocity of approximately 8 m/s for 5 min, NEs prepared with ultrasonication with an amplitude of 20%, a pulse mode of 10/10 using an ice bath. Each value on the plot represents the average and standard deviation of 3 replicates.

NEs were prepared under more challenging conditions, such as 20% amplitude and the use of an ice bath, to make the possible surfactant effects more evident. Three oil phases with low to medium viscosity, EtO, MCT, and SeO, were used, while the high-viscosity oils were excluded as they were not suitable for NE formation under these conditions. The results of the NE characteristics as a function of surfactant type are reported in Figure 6. Notably, the findings suggest that the NE’s Z_avg_ and PDI are largely independent of the surfactant type. These results align with the work of Pavoni et al. [17], who investigated NEs prepared by HPH, containing EtO and stabilized with the same amphiphiles used in this study. However, in the study by Pavoni et al. [17], the surfactants had a significant impact on NE stability. Although this aspect is not the primary focus of the present work, it was also examined. The results (an example is reported in Figure S7), seem to resemble previous findings, highlighting the following stability ranking: P80 > CS20 > CCG.

It is worth noting that these results and the observed independence of NE properties from the stabilizer are strictly bound to the investigated non-ionic surfactants. Given their similar HLB values and concentration ranges, these findings should not be broadly generalized to surfactants with different ionic characters, diverse molecular structures, or wider HLB variations.

#### 3.1.4. Oil Amount

**Figure 7:**
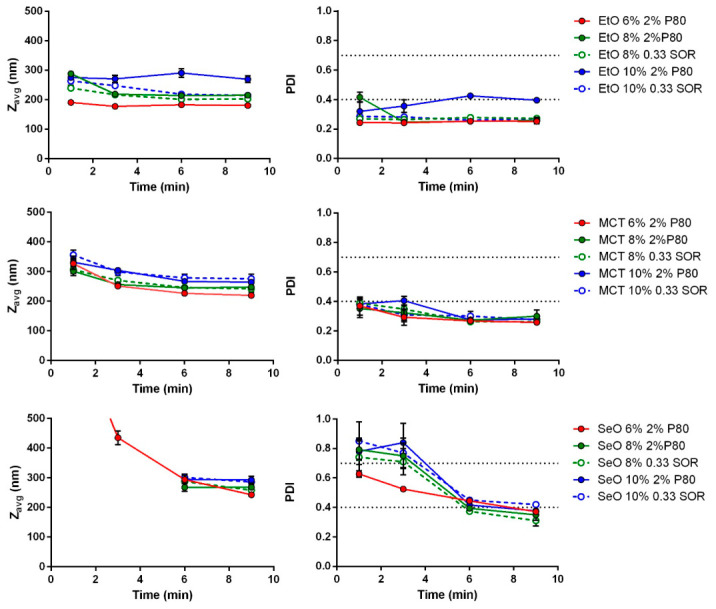
Effect of oil amount (6%, 8%, and 10%) on droplet Z_avg_ and PDI of NEs prepared with three different oil phases, EtO (**upper panels**), MCT (**middle panels**), and SeO (**lower panels**), keeping the surfactant (P80) constant at 2% or varying it to keep constant the SOR at 0.33. The NEs were prepared as follows: preliminary emulsion prepared operating at a linear velocity of approximately 8 m/s for 5 min, NEs prepared with ultrasonication with an amplitude of 30%, a pulse mode of 10/10 using an ice bath. Each value on the plot represents the average and standard deviation of 3 replicates.

The final set of experiments was designed to evaluate the effect of oil amount (8% and 10%, in addition to the 6% used so far) while operating either at a constant P80 concentration (2%) or increasing the surfactant proportionally to the oil phase (keeping a constant SOR of 0.33, corresponding to 2.66% and 3.33% of P80 for 8% and 10% oil, respectively). The NEs were prepared in an ice bath at 30% amplitude using EtO, MCT, and SeO as oil phases. The results (Figure 7) indicate that increasing the oil content from 6% to 10% leads to a slight average increase in PDI and Z_avg_, regardless of the surfactant amount in the formulation (2% or SOR 0.33). The effect of total oil amount in NE features appears to be related to oil viscosity and processing time. This is particularly evident for SeO, the highest-viscosity oil considered here, where the oil amount has the most pronounced impact at the shortest processing time, preventing NE formation. No clear trend is observed when increasing the surfactant concentration from 2% to a SOR of 0.33 (2.66% or 3.33%), suggesting that this parameter has no effect under the tested conditions.

### 3.2. General Analysis and Modelling with ANN

Given the high number of variables investigated, summarizing the results is challenging, as only one or few parameters were varied simultaneously for each group of experiments (for a total of more than 170 different experiments). Moreover, not all possible parameter combinations were tested, and the interpretation of overall trends is further complicated by variable interactions. To address these challenges, it was decided to identify a statistical tool capable of relating the experimental conditions to NEs’ formation and, if formed, to their Z_avg_ and PDI. A preliminary analysis compared the modelling ability of multilinear regression (MLR) and a multilayer perceptron (MLP) artificial neural network (ANN) in describing PDI. In both cases, the models were optimized by selecting the most suitable predictors for MLR and by adjusting the network architecture and the partition ratio between training and testing samples for MLP. The results indicate that both methodologies were able to describe PDI as a function of the input data; however, the determination coefficient of the MLP was significantly higher than that of the MLR (R^2^ of 0.883 and 0.795 for MLP and MLR, respectively, as reported in Figure S8). Additionally, MLR suffered from high collinearity among the input variables (as indicated by high variance inflation factors in the model coefficients analysis). Consequently, MLP was chosen as the preferred approach, not only due to its capacity to capture complex relationships within the data but also for its ability to better handle multicollinearity [29], leading to improved overall data description. The MLP was subsequently employed to model the Z_avg_ results. For this analysis, only the experiments yielding a PDI lower than 0.7 (approximately 150 tests) were included, as Z_avg_ results are considered unreliable for higher PDI values. In this case as well, the MLP demonstrated strong modelling capability, achieving an R^2^ of 0.911 (Figure S9). Details of the MLP model summary and the neural network architecture for both PDI and Z_avg_ are provided in the Supplementary Materials (Table S1 and Figure S10).

It is worth noting that while the ANN successfully mapped the complex, non-linear relationships of the non-factorial experimental space, its predictive validity is strictly limited to interpolation within the boundaries of the tested design space, and extrapolation to untested operational ranges should be avoided. Furthermore, because a partially sequential approach was adopted rather than a fully factorial design, we acknowledge that certain asymmetrical parameter combinations might introduce localized constraints or minor biases into the ANN sensitivity analysis. Nevertheless, the model remains highly robust in identifying the predominant macroscopic trends and the overarching hierarchy of the formulation and process variables within the investigated domain.

**Figure 8:**
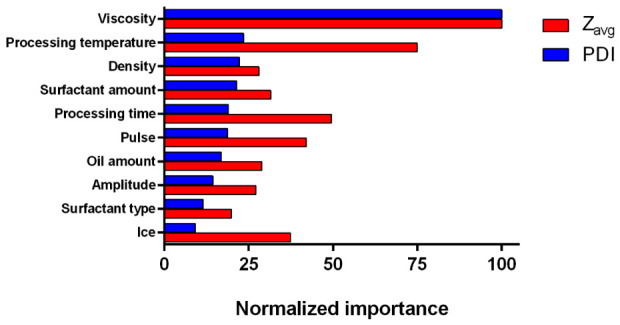
Sensitivity analysis performed on the two ANN models (Z_avg_ and PDI). The height of the bars represents the relative importance of each input variable with respect to the model’s predicted output. The most influential variable is set to 100%, and the importance of the other variables is expressed relative to this reference.

The MLP analysis allowed for the identification of the relative importance of the variables through a sensitivity analysis. This approach assessed the contribution of each predictor to the neural network’s performance, providing valuable insights into the most influential factors in determining the model’s outputs (PDI and Z_avg_). The sensitivity analysis results, reported in Figure 8, clearly indicate that while all variables influence the formation and features of NEs, certain factors stand out in their significance. Concerning the PDI, viscosity emerged as the most relevant parameter, with relative importance approximately four times higher (around 400%) than that of the second most important parameter, processing temperature. A similar trend was observed for Z_avg_, although the differences were less pronounced. In terms of absolute values, viscosity had a relative importance 50% higher than processing temperature. From a general point of view, all the other parameters had an influence lower than that of processing temperature for both PDI and Z_avg_. It should be noted that, except for viscosity, all experimental parameters tend to have a greater average impact on Z_avg_ than on PDI.

The predominant role of viscosity highlighted by the sensitivity analysis can be interpreted within the framework of droplet breakup dynamics during ultrasonication. Acoustic cavitation generates intense local shear fields, microjets, and shock waves that promote droplet deformation and fragmentation. The ability of these stresses to overcome interfacial forces is commonly described by the Weber number, whereas the resistance of the dispersed phase to deformation is quantified by the Ohnesorge number (Oh). According to the scaling framework proposed by Gupta et al. [11], NE formation typically occurs in a high-Oh regime, where internal viscous stresses within the droplets cannot be neglected. Under these conditions, the critical Weber number required for droplet breakup increases with viscosity, meaning that stronger disruptive forces are needed to achieve fragmentation. As a result, a larger fraction of the ultrasonic energy delivered through cavitation is dissipated within the droplets rather than being used for droplet breakup. This mechanism explains why viscosity emerged in the present work as the dominant variable affecting both Z_avg_ and PDI. Higher viscosities reduce the efficiency of droplet fragmentation, leading to larger droplet sizes and broader size distributions.

**Figure 9:**
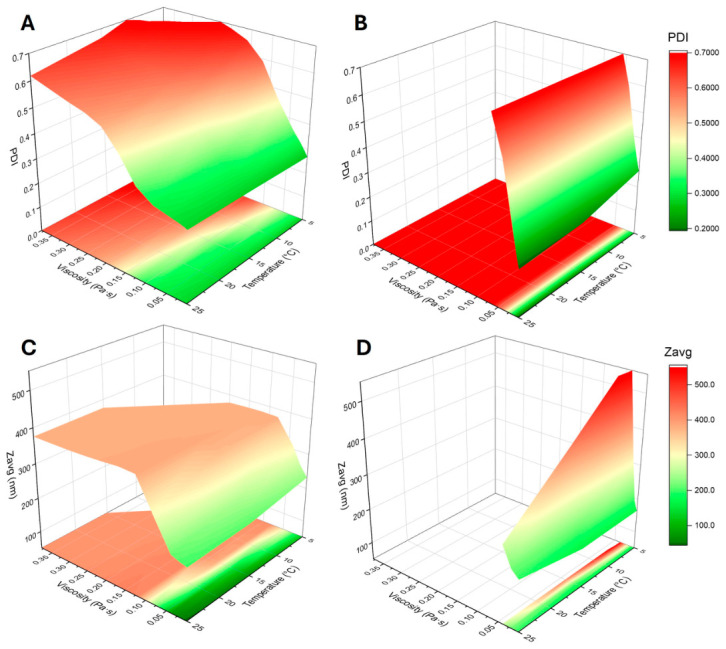
Predicted effect from the ANN modelling of oil viscosity and processing temperature on (**A**) PDI of NEs prepared under the favorable conditions; (**B**) PDI of NEs prepared under the unfavorable conditions; (**C**) Z_avg_ of NEs prepared under the favorable conditions; (**D**) Z_avg_ of NEs prepared under the unfavorable conditions. The absence of colors for some combinations of oil viscosity and processing temperature for Z_avg_ indicated experimental domains not modelled due to unsuitable values of PDI (>0.7).

Additionally, the MLP model was used to predict NEs’ features. Given the high number of variables and the limitation of visualizing at most two varying parameters in a 3D plot (with two predictors and one response), predictions focused on evaluating the effect of the two most relevant parameters, viscosity and temperature. All other variables were fixed at two defined levels: favorable conditions and unfavorable conditions. The terms favorable and unfavorable conditions do not represent the absolute best and worst conditions (i.e., the optimal and least favorable settings across all possible parameter combinations) but rather refer to common conditions used for NE preparation. In both setups, NEs were prepared with an oil concentration of 6% and P80 as the surfactant at 2% concentration and pulse ratio of 10:10. In the favorable conditions setup, all the other operative parameters were fixed at the most suitable values according to the model: amplitude at 30%, processing time of 9 min, low-density oil, and operating at ambient temperature. In the unfavorable conditions setup, the parameters were set to their least suitable values (amplitude at 20%, processing time of 3 min, high-density oil, and use of the ice bath). A processing time of 3 min was chosen because 1 min is never used in standard practice. The results, shown as surface plots in Figure 9, clearly illustrate the role of viscosity in determining both the feasibility of obtaining NEs and their features.

An important aspect is not only to identify the general conditions under which NEs can be formed, but also to define the specific parameters that characterize NEs with the desired features. In a broad sense, NEs are commonly defined as emulsions with submicron droplet size [6,30]. However, proper Z_avg_ and PDI values may vary depending on the application or even on the scientific field considered, and there is no universal consensus in the literature regarding the optimal size range. In this work, we define as “proper” NEs those with relatively small droplet size and moderately low polydispersity, specifically with a PDI below 0.4 and a Z_avg_ below 300 nm. These values were chosen because they proved suitable for our applications.

The data in Figure 9 highlight that achieving proper NEs (shown in yellow and green color in Figure 9) is primarily constrained by Z_avg_, which requires more narrowly defined conditions compared to those needed to achieve a PDI of 0.4. Model predictions also allow for the definition of a critical viscosity, above which the production of proper NEs is not possible, while for lower values, NEs’ formation and features are influenced also by the other factors (such as temperature, amplitude, etc.). Since Z_avg_ is the limiting response, the critical viscosity has been determined from its predictive model, ranging from 0.080 to 0.033 Pa·s (depending on temperature) under the favorable conditions setup and from 0.046 to 0.018 Pa·s (depending on temperature) under the unfavorable conditions setup. According to these critical viscosity values, proper NEs can be prepared under almost all conditions when using EtO and IsoM as the oil phase, given their power-law viscosity of approximately 0.0033 Pa·s; MCT is also a viable option, as its power-law viscosity (around 0.02 Pa·s) is very close to the lowest limit in the unfavourable conditions setup. For SeO and EVO (power-law viscosity of around 0.04–0.05 Pa·s), proper NEs can be prepared by setting precise operating conditions, while PaO and SiO (power-law viscosity > 0.15 Pa·s) are unsuitable as oil phases in NEs. It should be noted that the thresholds of Z_avg_ < 300 nm and PDI < 0.4 are operational criteria commonly used in many NE applications. While applying stricter constraints would shift the calculated critical viscosity values, the overall non-linear trends and the predictive architecture established by the model remain valid. As an example, by applying the stricter conditions commonly required for intravenous administration (Z_avg_ < 200 and PDI < 0.3), the most favorable critical viscosity values drop to a narrow range from 0.017 to 0.004 Pa·s in the temperature range 15–25 °C (and the NEs cannot be achieved at lower temperature). This sharp decrease is directly related to the local steep slope of the model’s response surface (Figure 9C).

## 4. Conclusions

This study evaluated the impact of oil viscosity, surfactant type, processing parameters, and formulation composition on the formation of oil-in-water NEs prepared via HEU. Among all factors, oil viscosity was identified as the most critical determinant affecting the NE formation, with high-viscosity oils leading to large Z_avg_ and high polydispersity or even failed nanoemulsification. This effect was found to be independent of pre-sonication droplet size, confirming the direct role of viscosity during the ultrasonication process. Processing temperature, whether controlled passively (via an ice bath) or actively (via pulse modulation), significantly impacted NE features by modulating viscosity during processing, although a more complex interplay of physical phenomena involving cavitation dynamics and interfacial properties could likely be involved. Droplet Z_avg_ and distribution, particularly under challenging conditions when working with high-viscosity oils, were also affected by ultrasound amplitude and pulse mode, while rising oil concentrations led to modest increases in both PDI and Z_avg_, with more pronounced effects at shorter sonication times and for higher-viscosity oils. Results suggest that the Z_avg_ and PDI of NEs are largely independent of the surfactant type; however, this finding is strictly limited to the investigated non-ionic surfactants (P80, CS20, and CCG) and cannot be generalized to stabilizers with different ionic characters or broader HLB ranges. The use of MLP artificial neural networks enabled the modelling and prediction of NE characteristics with high accuracy, outperforming traditional multilinear regression. Sensitivity analysis further confirmed the dominant role of viscosity, followed by processing temperature, in determining NE features. The model also enabled the definition of critical oil viscosity thresholds, beyond which the formation of proper NEs (defined as Z_avg_ < 300 nm and PDI < 0.4) becomes unfeasible using HEU, at least under the applied processing conditions examined in this work. In conclusion, this work provides a robust framework for the rational design of nanoemulsion formulations via HEU, demonstrating the value of predictive modelling tools for guiding formulation development in complex multi-parameter systems. These formulation insights can have a significant impact in all technological sectors (pharmaceutical, food, and agriculture), in which NEs offer valuable real-world applications.

## Data Availability

Data is contained within the article or Supplementary Materials.

## Supplementary Materials

The following supporting information can be downloaded at: https://www.mdpi.com/article/10.3390/pharmaceutics18070786/s1, Figure S1: Average flow curves (A), power law viscosity (B), power law index (C) and density of the different oils tested. The bars represent the medium value, while the error bars the standard deviation; Figure S2: Droplet size distribution of the preliminary emulsions prepared with different oils using a high-energy rotor–stator disperser equipped with an 8 mm diameter rotor, operating at 9500 rpm for 5 min. The horizontal line within the box represents the median Feret diameter (D50), while the lower and upper edges of the box correspond to the first (Q1) and third (Q3) quartiles of the droplet size distribution. The whiskers indicate the range covering the bottom 25% and top 25% of the data values; Figure S3: Correlation analysis between the median Feret diameter (D50) and oil viscosity (left panel) and oil density (right panel); Figure S4: Correlation analysis between the median Feret diameter (D50) and the hydrodynamic diameter (Z_avg_) (left panel) and polydispersity (PDI) (right panel) of the resulting nanoemulsions. All nanoemulsions were prepared using the ‘initial’ HEU conditions defined in Section 2.2.3 (Nanoemulsion Preparation); Figure S5: Droplet size distribution of the preliminary emulsions prepared with isopropyl myristate, sesame oil, and paraffin oil using a high-energy rotor–stator disperser. The emulsification conditions were adjusted to achieve a nearly comparable droplet size distribution; Figure S6: Effect of different pulse settings at ambient temperature or in an ice bath on the temperature increase during ethyl oleate NEs preparation; Figure S7: Effect of different surfactants at 2% on the size of 6% MCT NEs prepared at 20% amplitude, pulse ratio 10:10 using an ice bath. The continuous lines represent the curve trends obtained after smoothing the raw data only to help the readability of the plot; Figure S8: Experimental vs. predicted PDI (with the corresponding coefficient of determination) obtained using the best multiple linear regression (MLR) model and the multilayer perceptron (MLP) approach; Figure S9: Experimental vs. predicted PDI (with the corresponding coefficient of determination) obtained using the multilayer perceptron (MLP) approach; Figure S10: MLP architecture for PDI (A) and Z_avg_ (B); Table S1: Model summary for MLP modelling of PDI (left box) and Z_avg_ (right box).
