# Supplementary material for: Unveiling the Role of Formulation and Process Variables in Nanoemulsion Preparation: A Data-Driven Approach Using High-Energy Ultrasonication
Source: Pharmaceutics. 2026 Jun 26;18(7):786. doi: 10.3390/pharmaceutics18070786 (PMC13415607; doi:10.3390/pharmaceutics18070786)
Supplement: Supplementary file 1 [file pharmaceutics-18-00786-s001.zip › pharmaceutics-4348225-supplementary.pdf]

Supplementary materials of

Optimizing Nanoemulsions Formulation and Preparation: A Data-Driven Approach Using High- Energy Ultrasonication

Diego Romano Perinelli[1], Ledjan Malaj[2], Laetitia Novelli[1], Marco Cespi[1]* and Giulia Bonacucina[1]

1. Chemistry Interdisciplinary Project (ChIP) building, School of Pharmacy, University of Camerino, Camerino, Italy; marco.cespi@unicam.it ;diego.perinelli@unicam.it; giulia.bonacucina@unicam.it; laetitia.novelli@unicam.it
2. Department of Pharmacy, University of Medicine Tirana, Tirana, Albania; ledjan.malaj@umed.edu.al

*Correspondence: marco.cespi@unicam.it

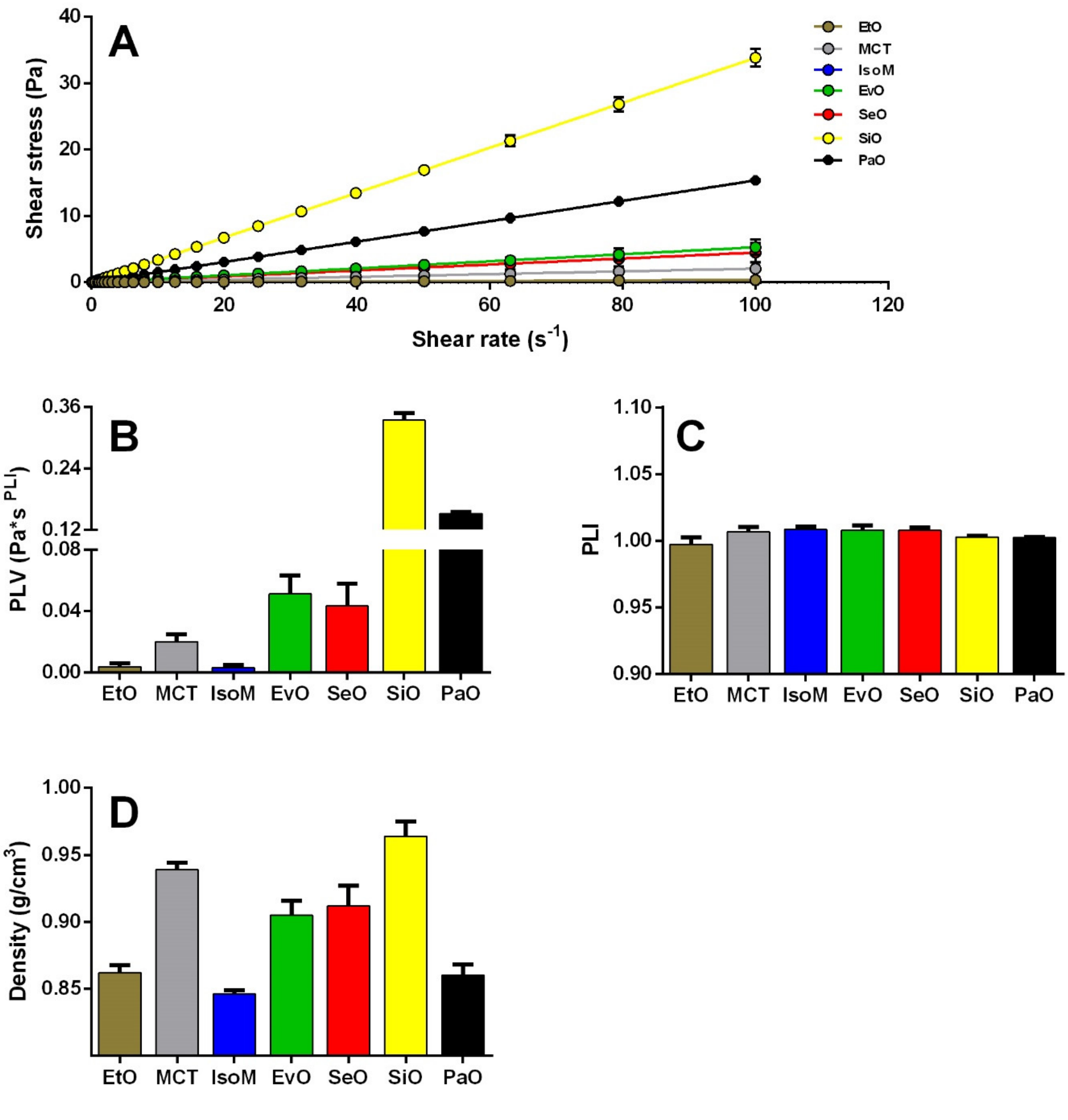

Figure S1: Average flow curves (A), power law viscosity (B), power law index (C) and density of the different oils tested. The bars represent the medium value, while the error bars the standard deviation.

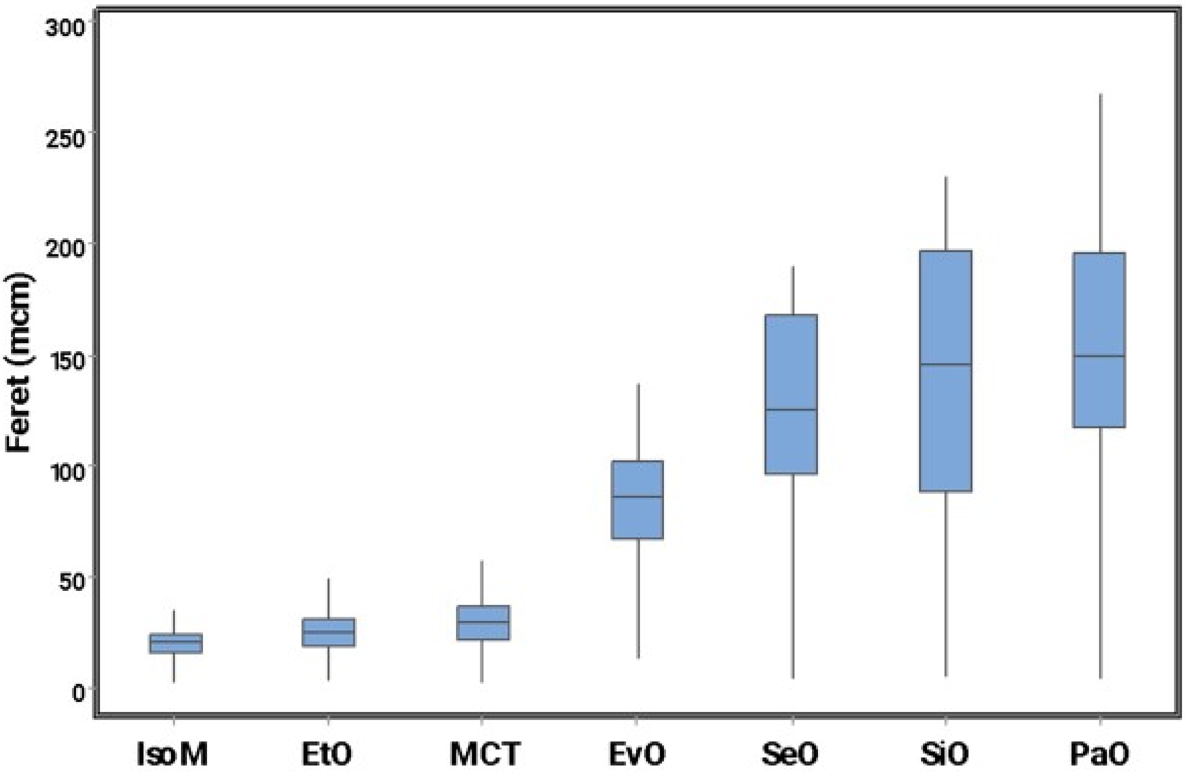

Figure S2: Droplet size distribution of the preliminary emulsions prepared with different oils using a high-energy rotor-stator disperser equipped with an 8 mm diameter rotor, operating at 9500 rpm for 5 minutes. The horizontal line within the box represents the median Feret diameter (D_{50}), while the lower and upper edges of the box correspond to the first (Q1) and third (Q3) quartiles of the droplet size distribution. The whiskers indicate the range covering the bottom 25% and top 25% of the data values.

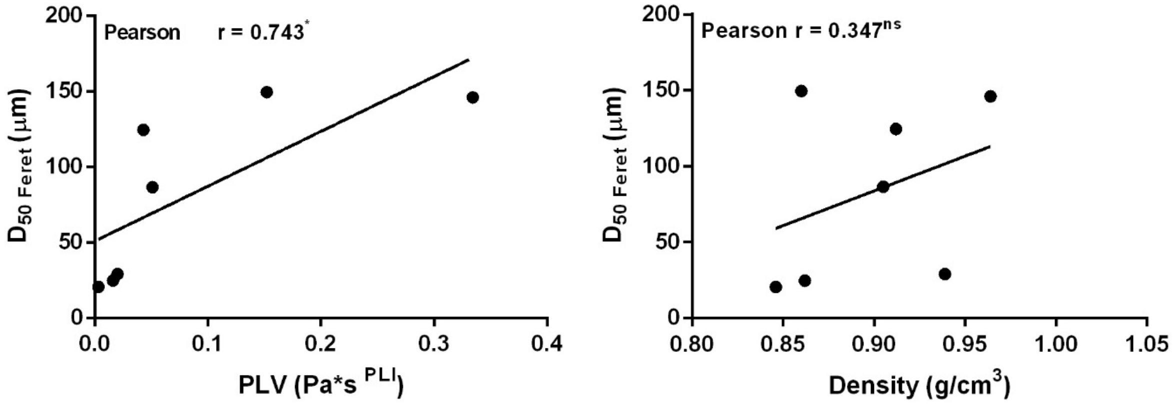

Figure S3: Correlation analysis between the median Feret diameter (D_{50}) and oil viscosity (left panel) and oil density (right panel).

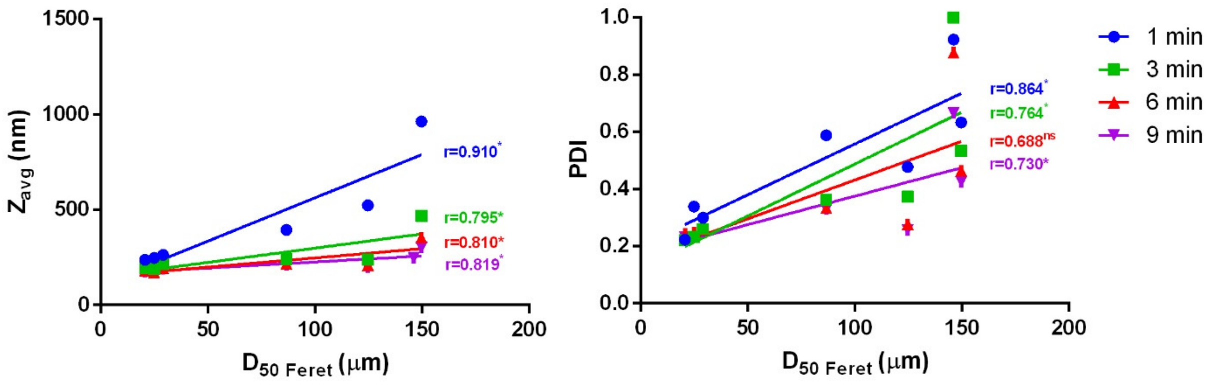

Figure S4: Correlation analysis between the median Feret diameter (D50) and the hydrodynamic diameter (Z_{avg}) (left panel) and polydispersity (PDI) (right panel) of the resulting nanoemulsions. All nanoemulsions were prepared using the 'initial' HEU conditions defined in Section 2.2.3 (Nanoemulsion Preparation).

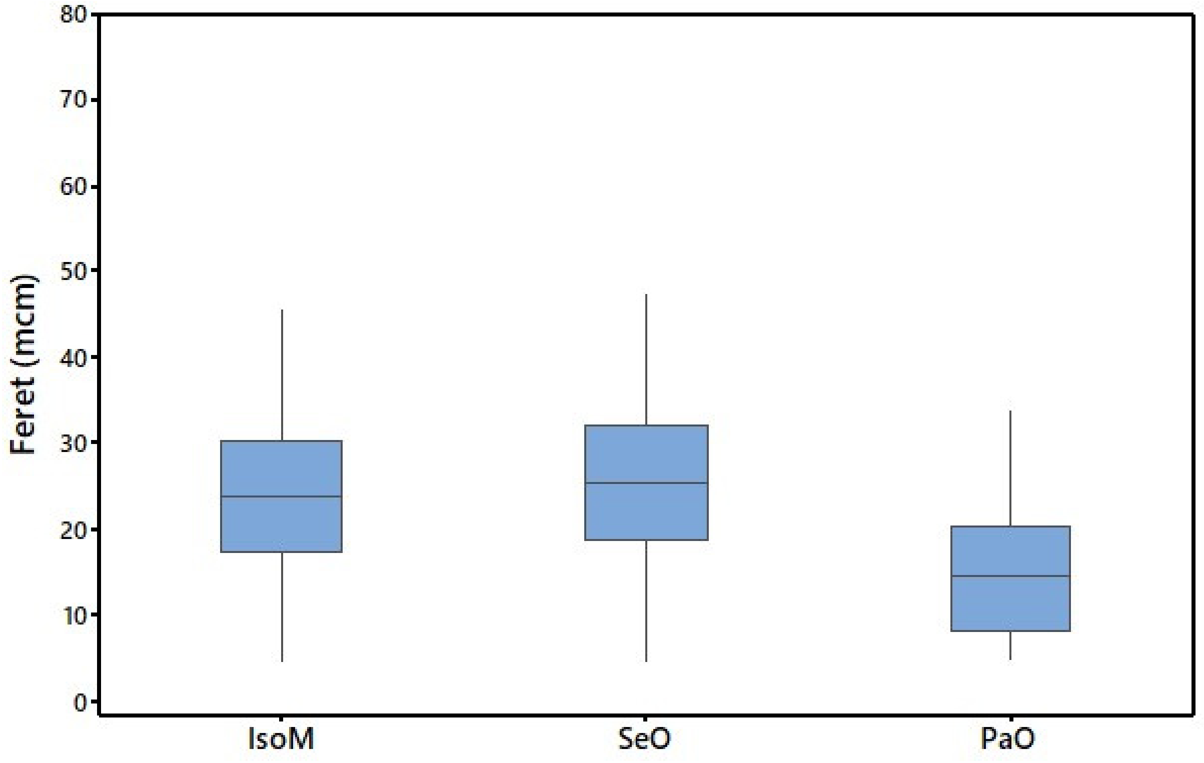

Figure S5: Droplet size distribution of the preliminary emulsions prepared with isopropyl myristate, sesame oil, and paraffin oil using a high-energy rotor-stator disperser. The emulsification conditions were adjusted to achieve a nearly comparable droplet size distribution.

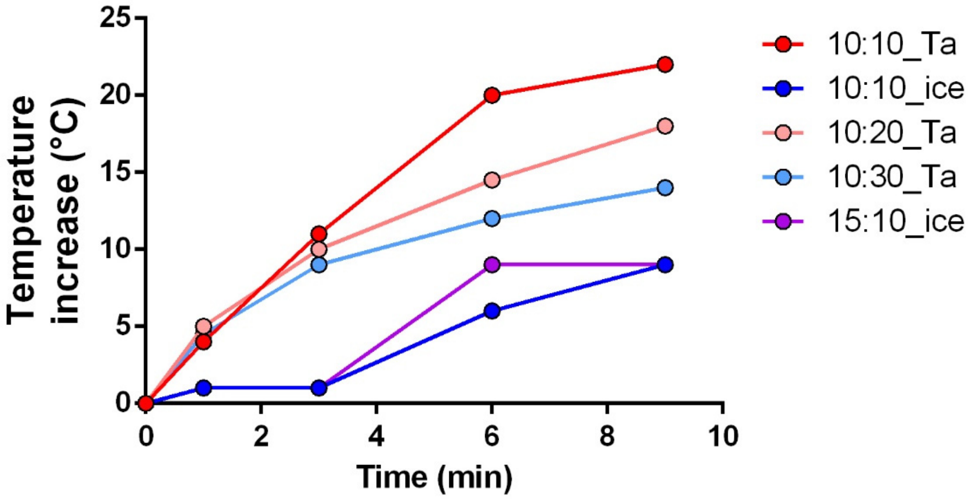

Figure S6: Effect of different pulse settings at ambient temperature or in an ice bath on the temperature increase during ethyl oleate NEs preparation.

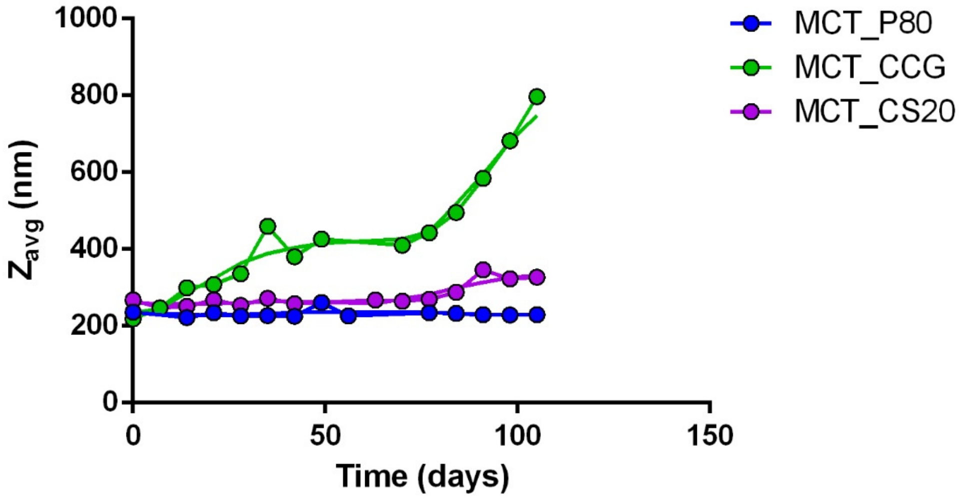

Figure S7: Effect of different surfactants at 2% on the size of 6% MCT NEs prepared at 20% amplitude, pulse ratio 10:10 using an ice bath. The continuous lines represent the curve trends obtained after smoothing the raw data only to help the readability of the plot.

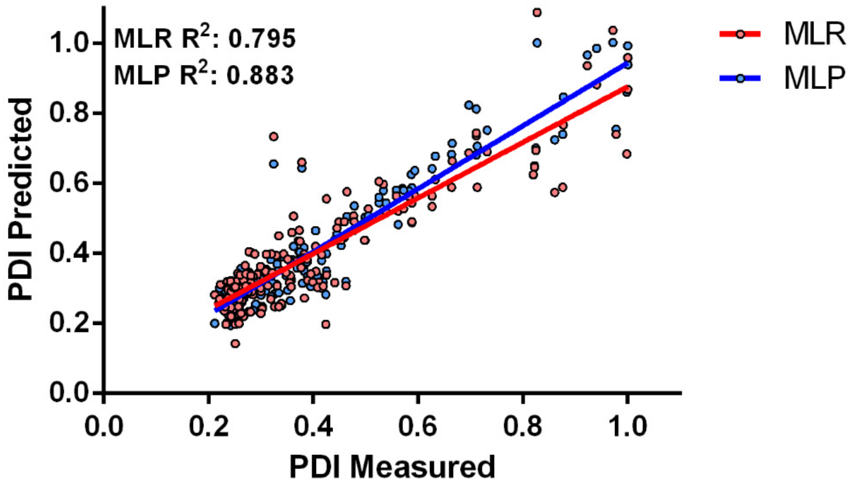

Figure S8: Experimental vs. predicted PDI (with the corresponding coefficient of determination) obtained using the best multiple linear regression (MLR) model and the multilayer perceptron (MLP) approach.

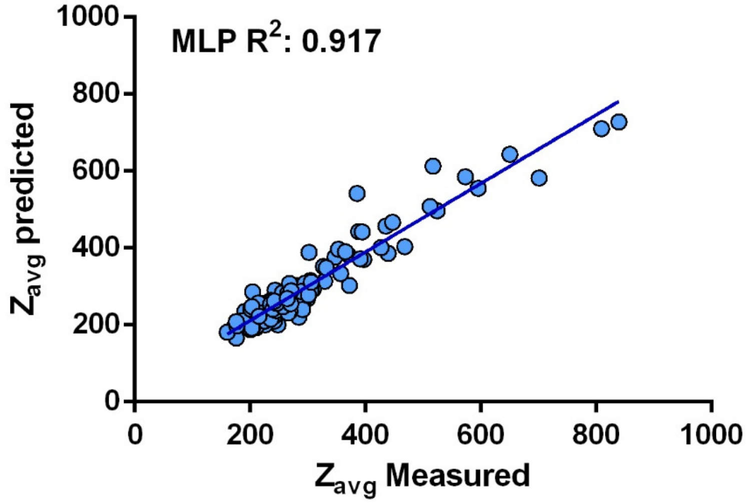

Figure S9: Experimental vs. predicted PDI (with the corresponding coefficient of determination) obtained using the the multilayer perceptron (MLP) approach.

Table S1: Model summary for MLP modelling of PDI (left box) and Z_{avg} (right box).

Model Summary (left box)

Training	Sum of Squares Error	6,478
	Relative Error	,097
	Stopping Rule Used	1 consecutive step(s) with no decrease in error[a]
	Training Time	0:00:00,03
Testing	Sum of Squares Error	3,502
	Relative Error	,199

Dependent Variable: PDI

a. Error computations are based on the testing sample.

Model Summary (right box)

Training	Sum of Squares Error	5,431
	Relative Error	,086
	Stopping Rule Used	1 consecutive step(s) with no decrease in error[a]
	Training Time	0:00:00,11
Testing	Sum of Squares Error	1,697
	Relative Error	,082

Dependent Variable: Z_avg

a. Error computations are based on the testing sample.

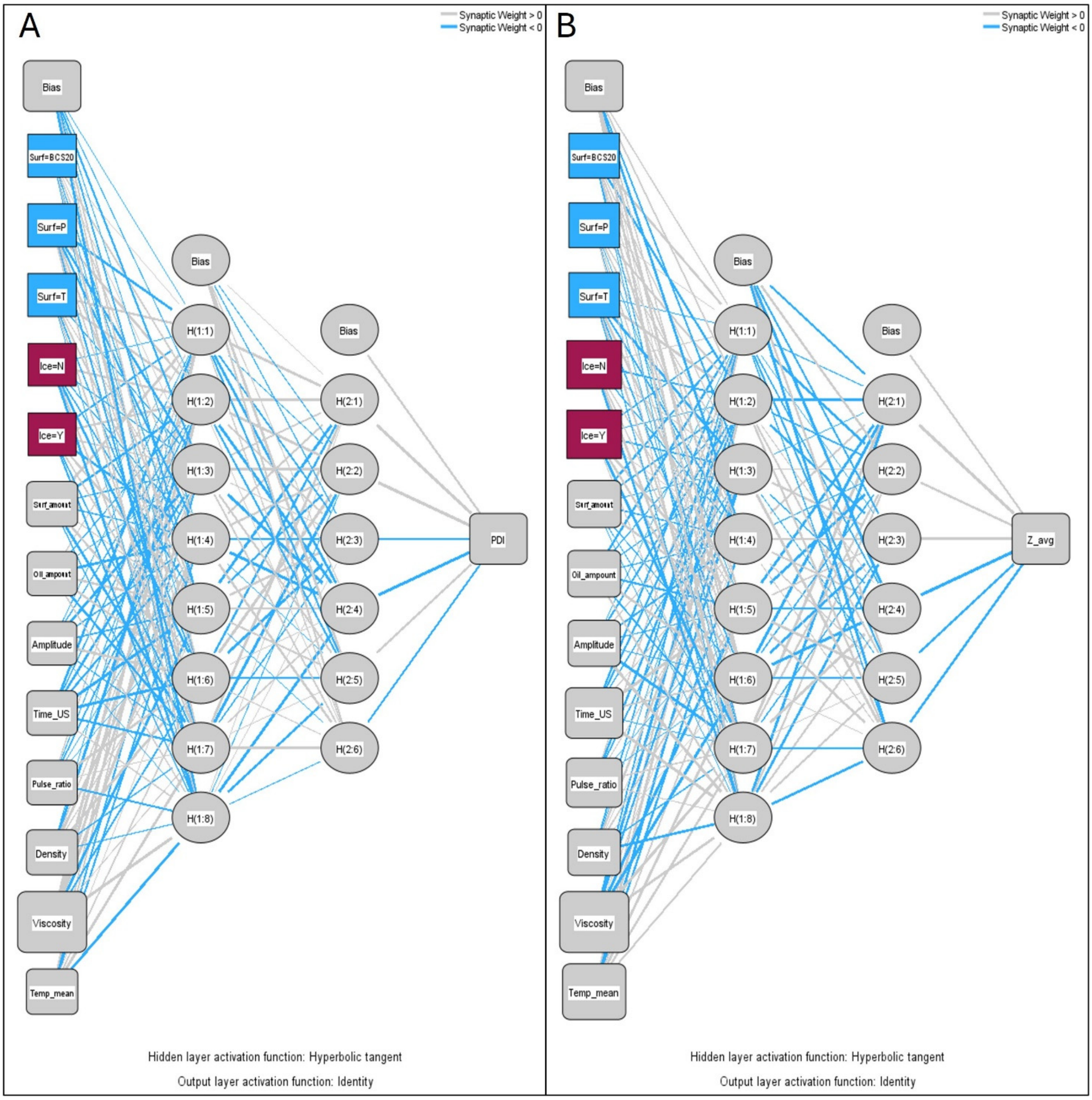

Figure S10: MLP architecture for PDI (A) and Z_{avg} (B).
